# Stepwise approach to establishing multiple outreach laboratory information system-electronic medical record interfaces

**DOI:** 10.4103/2153-3539.63829

**Published:** 2010-05-26

**Authors:** Liron Pantanowitz, Wayne LaBranche, William Lareau

**Affiliations:** 1 Department of Pathology, Baystate Medical Center, Tufts University School of Medicine, Springfield, MA, USA; 2 Department of Information Services, Baystate Medical Center, Tufts University School of Medicine, Springfield, MA, USA

**Keywords:** EMR, information system, interface, laboratory, LIS, outreach, SAAS

## Abstract

Clinical laboratory outreach business is changing as more physician practices adopt an electronic medical record (EMR). Physician connectivity with the laboratory information system (LIS) is consequently becoming more important. However, there are no reports available to assist the informatician with establishing and maintaining outreach LIS–EMR connectivity. A four-stage scheme is presented that was successfully employed to establish unidirectional and bidirectional interfaces with multiple physician EMRs. This approach involves planning (step 1), followed by interface building (step 2) with subsequent testing (step 3), and finally ongoing maintenance (step 4). The role of organized project management, software as a service (SAAS), and alternate solutions for outreach connectivity are discussed.

## INTRODUCTION

Many hospital laboratories have developed or are enhancing their outreach programs (i.e. performing laboratory services for the non-inpatient) to increase their test volumes, utilize excess capacity (i.e. improve productivity), bring on esoteric tests in-house, and consequently ease their financial burden.[1–3] It is estimated that around 90% of hospitals in the USA have some type of laboratory outreach program.[4] Clinical laboratory outreach business appears to be increasing as more physician practices adopt an electronic medical record (EMR). Understandably, physicians want to access their patient's results and electronically order laboratory tests within their own EMR. As a result, client connectivity with a legacy laboratory information system (LIS) is becoming more important in competitive environments.[5,6] Electronic health information exchange is a key component of competing effectively in the laboratory outreach market.

Much attention has been focused on important business strategies aimed at creating and maintaining a successful outreach program. Some of these tactics include aggressive marketing, hiring of a focused sales team, staffing client services, competitive pricing, seeking out managed care affiliations, offering timely and appropriate testing services, providing convenient courier services and last, but not the least, deploying a sophisticated LIS.[7] However, there are no published guidelines for informaticians to follow in order to assist their laboratories in establishing and maintaining outreach LIS–EMR interfaces. This technical note provides a stepwise approach, based upon experience at one institution, to successfully interface an LIS with multiple regional EMRs.

## TECHNICAL BACKGROUND

**Figure 1:**
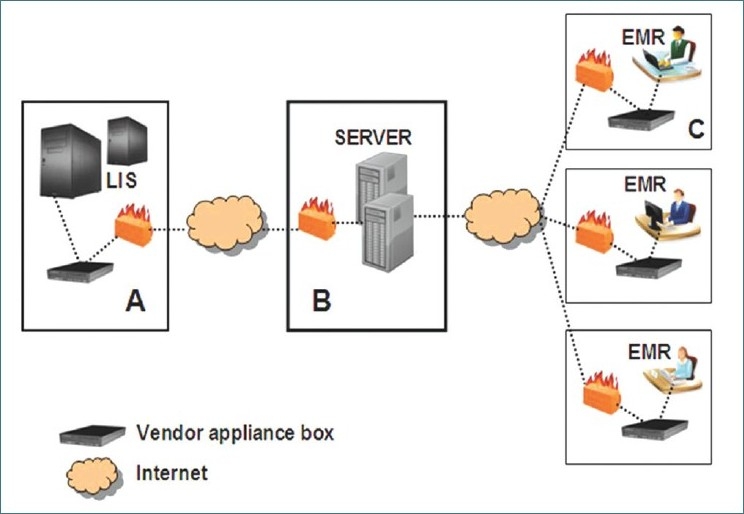
Visual depiction of data transmission between the LIS and outreach physician practice EMRs using SAAS. (A) Within the hospital, data from the LIS are transmitted via an interface engine to the vendor's appliance box. (B) Data are securely transmitted over the internet to the vendor's broker web server in their datacenter. These servers manage the routing and handle any data translation that may be required. Once complete, the data are again securely transmitted over the internet to the practice communication device that sits behind the practice's firewall. (C) The vendor enables communication and transmits electronic messages between their appliance box and the physician practice (client) EMR.

A separate Clinical Pathology LIS (Sunquest version 6.2, Sunquest Information Systems, Tucson, AZ, USA) and Anatomical Pathology LIS (CoPath version 3.1, Cerner Corporation, Kansas City, MO, USA) were utilized by Baystate Reference Laboratories (BRL; Springfield, MA, USA). Many regional physician practices referring their laboratory testing to BRL had acquired a wide variety of EMRs (including eClinicalWorks, MediNotes, SOAPware, NextGen, Script Sure, Sage Medical manager, SSIMED EMRge, Allscripts, Renal Track, ePro, Athena Health, and Practice Partner). In addition to relying on laboratory and hospital information services technical staff, a software as a service (SAAS) business model was employed using Initiate (an IBM company) Exchange platform with additional exchange service components, including Master Data Synchronization Service and TXM Bidirectional Reconciler. Transmission of data in this health information exchange model is illustrated in Figure 1. Data exchange consisted of two components: (a) appliance boxes called Initiate Lynx which are physically located at each end of the data feed and (b) servers at Initiate's datacenter. The Initiate Lynx communication device contains a Linux-based operating system within a small form factor personal computer about the size of a home cable box. Therefore, the laboratory had online access to outreach software (e.g. Master Data Synchronization Service) to build and maintain LIS–EMR interfaces. Initiate also provided assistance with technical operations, implementation and 24 × 7 software and service support by means of a complete monitoring toolset that proactively identifies integration type error conditions along the health exchange network to and from all practices. Data to and from the LIS was transmitted via the hospital's interface engine (SUN eGate) through a firewall to the SAAS vendor's server, from where the converted and remapped data were deployed to all physician EMRs.

## STEPWISE APPROACH

A four-stage scheme was employed to establish unidirectional and bidirectional interfaces with physician practice EMRs. This approach involved planning (step 1), followed by interface building (step 2) with subsequent testing (step 3), and finally ongoing maintenance (step 4).

*Step 1*: The initial planning phase included finance (budget), infrastructure, test volume (tests/year/practice) and backload parameter analysis. “Backload” refers to archival data clients requested to be added to their EMR, determined by performing a retrospective LIS data review specific for each physician client practice. Archival LIS data up to 2 years back were transmitted to select EMRs. At this stage, the identification of resources, roles, and responsibilities was carried out, and a schedule determined.

*Step 2*: In several EMRs, the test names and codes did not match those used by the LIS. In order to address this discrepancy, the so-called build phase involved the creation of a test compendium for bidirectional interfaces using IBM's Initiate Exchange software, specifically the Master Data Synchronization Service. The test compendium is a practice-specific translation table matching test codes and nomenclature between the computerized physician order entry (CPOE) module in the EMR and those in the LIS. The compendium mapped both order codes [Table 1] and result codes [Table 2] in the HL7 message. As unidirectional interfaces involved only result reporting, no such compendium was required. As no virtual private networks (VPN) were involved in this configuration, all clients required internet connectivity before appliance boxes, as depicted in Figure 1, and this was installed at their end behind the practice's firewall.

**Table 1:** Example of the test compendium for order matchinga^a^

SQ Code	Description (OBR-4.2)	Alias (OBR-4.1)	CPT
STLFAT	Stool for fat	64625	82705
UEOS	Urine EOS	64475	81015
UMICRO	Urine microscopic	64430	81015
FATST	Fat stain	64275	82705
APT	APT test	64225	83033
UIODQ	Urine iodine	64200	83789
USGR	Specific gravity	64185	81003
UALB	Urine albumin	64180	81003
UPH	Urine pH	64175	81003

a This was utilized for EMRs that required matching of the order alias (within OBR segment) in the HL7 message with the Sunquest (SQ) code and Current Procedural Terminology (CPT) code.

**Table 2:** Example of the test compendium for result matching^a^

SQ test Alias	Order name	Results #	SQ result Alias	Results description
ACETO	Acetone, blood	03005	ACE100	Acetone 100%
ACETO	Acetone, blood	03007	ACE50	Acetone 50%
ACETO	Acetone, blood	03009	ACE25	Acetone 25%
ACETO	Acetone, blood	03011	ACE10	Acetone 10%
ACETO	Acetone, blood	03013	ACE1	Acetone 1%
ACETO	Acetone, blood	03014	ACTONE	Acetone
TOPMAX	Topamax	03039	TOPMX	Topamax
GENTPK	Gent peak	03053	GENTPK	Gent peak
GENTTR	Gent trough	03058	GENTTR	Gent trough
GENTRA	Gent random	03063	GENTRA	Gent random

a This was utilized mainly for EMRs that accepted data from the laboratory's SQ system and the client office's own laboratory system

*Step 3*: In the production phase, the laboratory was required to validate secure connectivity, match results to orders, adjust and endorse EMR lab data content and display (using electronic screenshots, EMR printed reports and/or GoToMeeting), as well as reconcile mismatched orders and/or results [Table 3]. The GoToMeeting web conferencing tool allowed the laboratory to securely collaborate online in real time with multiple remote EMR users, to view the display of laboratory data in the downstream EMR system. The TXM Bidirectional Reconciler was run on a dedicated Java 2 enterprise application server in Initiate's secure datacenter.

**Table 3:** Example of the reconciler utilized for bidirectional interface monitoring^a^

Match%			MRN	Last name	First name	DOB	Sex	Account^#^	Requisition^#^	Order code	Order MD	Order date
29%	Lab data	Result	1111111	Patient	Katharine	02/29/1950	F	801168301	648038005	54650	Doctor, John	08/22/2009
	Client data	Order	2222222	Patient	Fredrick	01/03/1951	M		2609332	54650	Doctor, John	08/21/2009

a The reconciler only shows mismatched transmissions that need to be reconciled. DOB: Date of birth; F: Female; M: male; MRN: Medical record number

*Step 4*: For this monitoring phase, a prior service-level agreement with clients was required, along with a downtime procedure, connectivity monitor, monitoring of EMR and LIS error capture logs, mechanisms to continually check the display of EMR lab data and update the test compendia, as well as a change control procedure for potential software upgrades.

## CONCLUSION

There are several take-home messages from the aforementioned approach used to connect the LIS with multiple disparate EMRs. First, a project management approach is fundamental. Proper planning, organization, and management of resources are necessary to successfully complete any project. Developing an outreach program may require additional resources and alignment of technical efforts with business goals. Second, SAAS in this instance formed a vital component of establishing and maintaining laboratory outreach connectivity. SAAS is a model of software deployment whereby the provider (Initiate in this case) offers a full service solution to customers (our laboratory) for use as a service on demand.[8] In this model, the SAAS vendors host their application on their own servers, providing web-based access to and management of their remote software. The SAAS vendor further provides and supports all hardware, including communication devices on the entire exchange platform. This scalable model permitted the laboratory to focus their budgets on competitive rapid deployment, rather than infrastructure.

Physician connectivity can be accomplished directly through the LIS or hospital information system using an interface engine platform or by means of a separate outreach “wraparound” (kludge) system.[3] The approach described in this technical note provides an example of such a wraparound system. While establishing connectivity directly with the laboratory hospital may take longer and taxes hospital IT resources, this solution offers the client access to all patient medical record information. On the other hand, while specialized wraparound systems may require additional funding, they usually offer more capabilities (e.g. interface reconciler, generation of advanced beneficiary notice forms, etc.) and can support more rapid creation of new connections. Other solutions to connect remote practices with a laboratory include secure web-based portals, allowing clients to submit orders and receive results via the Internet.[9] Physician connectivity with the laboratory often facilitates an infrastructure to establish electronic exchange of all health information (e.g. radiology, cardiology, etc.). This is imperative for retaining clients because physician practices are particularly interested in integrating their EMR to as much clinical information in the patient record as possible. Finally, enhanced LIS features that better support outreach programs,[10] interoperability standards, and improved EMR vendor cooperation are essential for electronically integrating healthcare.
